# Development, Validity and Reliability of Sexual Health Measures for Spinal Cord Injured Patients in Iran

**Published:** 2013-07-31

**Authors:** Effat Merghati Khoei, Abbas Norouzi Javidan, Mahboobeh Abrishamkar, Mir Saeed Yekaninejad, Samira Chaibakhsh, Seyyed Hasan Emami-Razavi, Asie Mansouri, Koorosh Kamali, Tannaz Shoja, Marzieh Hajiaghababaei, Abolghasem Nikfallah

**Affiliations:** 1 Family & Sexual Health Division, Brain and Spinal Injury Research Center (BASIR), Tehran University of Medical Sciences, Tehran, Iran; 2 Iranian National Center of Addiction Study, Tehran University of Medical Sciences, Tehran, Iran; 3 Brain and Spinal Injury Research Center (BASIR), Tehran University of Medical Sciences, Tehran, Iran; 4 Urology Division, Brain and Spinal Injury Research Center (BASIR), Tehran University of Medical Sciences, Tehran, Iran; 5 Department of Epidemiology and Biostatistics, School of Public Health, Tehran University of Medical Sciences, Tehran, Iran; 6 Biostatistics and Epidemiology Division, Brain and Spinal Injury Research Center (BASIR), Tehran University of Medical Sciences, Tehran, Iran; 7 Department of Public Health, School of Public Health, Zanjan University of Medical Sciences, Zanjan, Iran

**Keywords:** Spinal Cord Injury, Sexual Health, Validity, Reliability

## Abstract

**Background:**

This study developed and validated a questionnaire to measure the sexual health of patients with spinal cord injuries (SCI).

**Materials and Methods:**

This was a cross-sectional study conducted at the Brain and Spinal Injury Research Center (BASIR), Tehran, Iran. Extensive review of literature, expert opinions, and encounters with SCI patients were used to develop and validate the questionnaires. There were 40 (32 males, 8 females) patients with SCI that presented for treatment at BASIR who enrolled in the study. Participants completed the questionnaires while they were admitted for medical care and during treatment follow-up visits. Participants completed the questionnaires twice, at a 2-4 week interval. Reliability testing for each measure was performed separately. Cronbach’s alpha was used for internal consistency and test-retest was used for reliability.

**Results:**

An expert committee approved the face and content validities of the questionnaires, Internal consistency of our questionnaires, was acceptable according to Cronbach’s alpha that ranged from 0.73 for the sexual activity measure to 0.90 for the sexual adjustment measure. Test-retest reliability was satisfactory. Intraclass Correlation Coefficient (ICC) of measures ranged from 0.65 for sexual function to 0.84 for sexual activity.

**Conclusion:**

The sexual health measures has provided a valid assessment of sexualityrelated matters in this sample of patients with SCI, which suggests that evaluation of sexual well-being may be useful in clinical trials and practice settings. Overall, the sexual health measures shows good internal consistency and test-retest reliability.

## Introduction

Spinal cord injury (SCI) is a common, debilitating physical condition. Data from the United States indicates that there are approximately 12000 new cases each year (1). The prevalence of SCI in Tehran, Iran is 4.4 (95% CI: 1.2-11.4) per 10000 people (2). Individuals with SCI experience physical and psychological problems that have a profound impact on their sexual health (3). Sexual despair adversely affects quality of life and interpersonal relationships of the SCI patient, as well as the partner (4). In fact, SCI is one of the main reasons for divorce. Of single patients with SCI, 90% remain unmarried after five years (5). In Iran this can be due to the common cultural perception, "those who cannot have sexual intercourse cannot get married". According to our experiences in working with Iranian couples with SCI, the marriage is typically sustained post-injury when the woman is the healthy partner.

To address the quality of sexual lives of SCI patients, a number of instruments have been developed (6).

The Emotional Quality of the Relationship Scale (EQR) was developed and validated by Kreuter et al. (7). This is a seven-item, self-report tool which measures the subjective meanings of sexuality. The Sexual Activity and Satisfaction scale (SAS) is a three-item, self-report tool used to investigate the sexual activity, sexual desire and sexual satisfaction of individuals with SCI. The Sexual Attitude and Information Questionnaire (SAIQ) evaluates the effectiveness of counseling programs and sexual education for people with SCI and their partners (8). Finally, the Sexual Interest and Satisfaction scale (SIS) is a seven-item scale, designed to measure sexual adjustment (9).

This study reported the sexual health measures developed and validated for Iranian individuals with SCI who presented to the Brain and Spinal Injury Research Center (BASIR) in Imam Khomeini Hospital. This research aimed to create culturally appropriate and practical tools that could be used to investigate sexual health among Iranian patients with SCI.

## Materials and Methods

Approval for this cross-sectional study was obtained from the Ethics Committee of Tehran University of Medical Sciences in 2009. Convenience sampling was used to recruit patients (N=68) from a larger study for health promotion of Iranian SCI patients. Participants completed the questionnaires while they were admitted for medical care and treatment follow-up. The written consent of participation was obtained prior to data collection.

To be included, participants had to be at least 18 years of age and have no medical diseases other than SCI that affected sexual health. Patients were evaluated based on the ASIA impairment scale (AIS) (10), which is a modification of the Frankel scale (11) that describes the degree of incompleteness below the level of a spinal cord lesion. The degree of incompleteness is graded on a 5-point scale from A to E. Most participants (42.5%) were categorized in C scale, followed by 25% (A), 17.5% (D) and 15% (B) as listed in table 1. Of these, 40 (32 male and 8 female) patients agreed to participate in the study. Written informed consent was obtained from participants prior to study participation.

The population on which questionnaires were tested comprised of two subgroups; 32 males aged 25-52 years (mean age: 35.69 ± 8.14) and 8 females aged 26-46 years (mean age: 34.29 ± 6.80). The mean age of all patients was 35.4 ± 7.8 years. Almost half (48.7%) of the participants were married. The average number of years of marriage was14.4 ± 8.5. The average number of years of SCI was 10.2 ± 8.5 years (Table 2).

**Table 1:** Internal consistency of the subscales and validation instruments


Measures	Cronbach's alpha

**Quality of social life**	0.887
**Sexual adjustment**	0.904
**Sexual activities**	0.730
**Sexual thoughts**	0.879
**Partnership satisfaction**	0.881
**Sexual function (male)**	0.901
**Sexual function (female)**	0.790


**Table 2:** Distribution of participants based on ASIA Impairment Scales (AIS)


		Frequency	Percent

**AIS**	A	10	25
	B	6	15
	C	17	42.5
	D	7	17.5
**Total**		40	100.0


### Development of the sexual health measures

As there were no existing sexual health questionnaires in the Farsi language to assess SCI patients, this study investigated six different multiple-choice questionnaires with a minimum of 7 to a maximum of 23 questions, which were designed in Farsi. Extensive literature review, opinions of 12 experts from various disciplines that included sexologists, urologists, epidemiologists, psychiatrists, midwifes, general practitioners and religious leaders, in addition to informal encounters with SCI patients were used to generate the instruments. Our intention was to develop a measure that could be used to assess sexual-related social life (questions 1-8), sexual adjustment after SCI (questions 9-19), sexual activities (questions 20-32), sexual thoughts (questions 33-39), performance partnership satisfaction (questions 40-55), and sexual functions (questions 56-65). Sexual-related social life characterizes the social component of a person’s sexuality, including interpersonal relationships.

In the current study, as with a study by Kreuter et al. (7) sexual adjustment refers to the psychological component of a person’s sexuality and includes sexual interest, satisfaction, selfesteem and feelings of interpersonal attractiveness. Sexual activities is defined by the behavioral component of the patients’ sexualities (i.e., any form of sexual interactions with or without intercourse that include physical contact such as kissing or petting). Sexual thoughts include imagining, perceptions, and memories with or without sexual arousal and performance. Partnership satisfaction are affectionate responses, either positive or negative that are related to a couple’s sexual relationship. The measures utilized a Likert-scale with the following five anchors: 1(strongly disagree), 2(disagree), 3 (undecided), 4 (agree), and 5 (strongly agree). The respondents reported their partnership satisfaction on a 1 to 7 Visual Analog Scale (VAS). Endpoints were labeled as 1(too bad) and 7 (too good). All questionnaire items, except those in the sexual function measure, were similar for male and female patients.

### Statistical analysis

The total score in each subscale was calculated by summing the items after which we transformed the total score using linear transformation

### Validity

Face validity, defined as whether the scale appears to measure what it is supposed to measure, and content validity, or the extent to which a measure comprehensively covers domains of interest were examined by an expert committee. Construct validity was assessed by examining convergent validity and hypothesizing that each sex subscale would be significantly negatively correlated with age and positively correlated with other subscales.

We computed Cronbach’s alpha to assess internal consistency of items as a function of the mean inter-item correlation among the six measures. A high Cronbach’s alpha value (>0.7), the standard criterion of acceptability, is the result of a high inter-item correlation which indicates that the items measure the same underlying construct (11, 12).

Correlation matrix based on Pearson’s product- moment correlation (Table 3) was used to analyze the test and re-test results. Test-retest reliability was employed to obtain external item reliability. The alpha-level was set at 0.05. Statistical analysis was performed using test-retest reliability of the instruments assessed by Intraclass Correlation Coefficient (ICC) at base line and at the end point. Measurements with ICC values between 0.6-0.8 suggested satisfactory stability, values between 0.8-0.9 were considered excellent, and values >0.9 were considered highly reproducible. (11).

To assess the test-retest reliability of the questionnaires, participants were asked to complete the questionnaire again, two to four weeks after initial participation. Reliability testing was performed separately for each measure.

**Table 3:** Test-retest reliability of the subscales by Intraclass Correlation Coefficient


Questionnaires	ICC	P value

**Quality of social life**	0.797	< 0.001
**Sexual adjustment**	0.825	< 0.001
**Sexual activities**	0.836	0.001
**Sexual thoughts**	0.784	0.003
**Partnership satisfaction**	0.787	0.001
**Sexual function (male)**	0.820	0.001
**Sexual function (female)**	0.653	< 0.001


## Results

The hypothesized significant negative correlation between the questionnaire subscales and age was supported. As expected, there was a significant positive correlation observed between subscales of the questionnaire (Table 4). The questionnaire, therefore, had convergent and construct validity.

### Reliability

#### Internal consistency

Internal consistency was determined using Cronbach’s alpha for each of the seven measures. High scores were eminent for all measures (0.73 and higher). Cronbach’s alpha ranged from 0.73 for the sexual activity measure to 0.904 for the sexual adjustment measure (Table 3).

#### Test-retest reliability

Test-retest reliability was determined using the ICC for each of the seven measures. ICCs ranged from 0.653 for sexual function (female) to 0.836 for sexual activity.

**Table 4:** Correlation of each sex domain with age and other domains


	1	2	3	4	5	6	7

**1. Age (Y)**	1	-0.22*	-0.18	-0.32	-0.15	-0.36	-0.45
** 2. Quality of social life**		1	0.68	0.53	0.69	0.48	0.46
**3. Sexual adjustment**			1	0.79	0.73	0.77	0.61
**4. Sexual activities**				1	0.75	0.64	0.67
**5. Sexual thoughts**					1	0.71	0.59
**6. Partnership satisfaction**						1	0.61
****							
**7. Sexual function**							1


All correlations were significant at the 0.01 significance level.

## Discussion

The primary purpose of the current study was to develop and validate sexual health measures for Iranian patients with SCI. Internal consistency was documented for each of the six measures. Cronbach’s alpha was 0.73 or higher which showed good internal consistency. Thus, it indicated that the items measured the same underlying construct. These high correlations were comparable to those reported by Meston (13) in all domains among women with female orgasmic dysfunction and control subjects (0.74 and higher).

However internal consistency in this study was lower than reported from some other studies. For example, Alexander et al. (14) reported a Cronbach’s alpha equal to 0.82 or higher. In another study by Wiegel et al. (15) Cronbach’s alpha ranged from 0.82 to 0.97.

ICC from test-retest reliability in the current study was 0.65 or higher which suggested satisfactory stability. The only exception was in female sexuality function measures, though ICC in the other measures was greater than 78%, which indicated near to excellent agreement. These results were comparable with ICC reported from other studies. Alexander et al. found an ICC between 0.79 and 0.86, in their study (14). However ICCs from this study were lower than reported in Wiegel’s study. In this study the ICC of test-retest reliability ranged from 0.79 to 0.88 (15).

The results suggested that a questionnaire with this level of sensitivity should be completed in male patients that have male assistants. Although we achieved significant results with this small sample size, we recommend that future studies be conducted with larger numbers of participants.

## Conclusion

The rehabilitation services do not have adequate, comprehensive sexual well-being programs in Iran. One reason for this deficit is the lack of culturally appropriate instruments to examine related variables and measure the interested outcomes among SCI patients.

Although a number of measures have been pubpublished concerning sexuality matters in SCI patients (6), we chose to develop our own questionnaire for a number of reasons that include linguistic obstacles; sexuality, as an unnoticed matter even at the academic level; and cultural dimensions of the existing tools.

To address this insufficiency we have aimed not only at the cognitive component of sexual health attitudes but also the behavioral component. Our study has shown that it was possible to construct measures with good psychometric characteristics regarding the quality of social life, sexual adjustment, sexual activities, sexual thoughts, partnership satisfaction and sexual function (males and females). The sexual health measures provided a valid assessment of sexuality-related matters in this sample of patients with SCI, which has suggested that it may be useful for evaluation of sexual well being in clinical trials. Overall, the studied sexual health measures have shown good internal consistency and test-retest reliability. This is the first study to validate a measure of sexuality-related matters on a sample of patients with SCI in Iran. Future research is needed to examine this measure in a larger study population.
